# Co-growth of Stem Cells With Target Tissue Culture as an Easy and Effective Method of Directed Differentiation

**DOI:** 10.3389/fbioe.2021.591775

**Published:** 2021-06-16

**Authors:** Marina Valentinovna Kovina, Tatyana Gennadievna Dyuzheva, Mikhail Evgenievich Krasheninnikov, Sergey Alexandrovich Yakovenko, Yury Mikhailovich Khodarovich

**Affiliations:** ^1^Peoples’ Friendship University of Russia, Moscow, Russia; ^2^AltraVita IVF Clinic, Moscow, Russia; ^3^The University of Texas Health Science Center at Houston, Medical School, Department of Integrative Biology and Pharmacology, Houston, TX, United States; ^4^Sklifosovsky Institute for Clinical Medicine, Sechenov University, Moscow, Russia; ^5^Department of Biophysics, M. V. Lomonosov Moscow State University, Moscow, Russia; ^6^Shemyakin-Ovchinnikov Institute of Bioorganic Chemistry of the Russian Academy of Sciences, Moscow, Russia

**Keywords:** differentiation, contact differentiation, embryonic stem cells, endothelial differentiation, co-culture, hepatic differentiation

## Abstract

The long-term co-culture of mouse embryonic stem cells (mESC) with rat endothelial cells (EC) was tested for contact differentiation into the endothelial lineage. Serial passaging of rat ECs mixed with mESC in ratio 10:1 resulted in the emergence of a homogeneous cell population expressing mouse endothelial surface markers CD102, CD29, CD31. Rat endothelial surface marker RECA-1 completely disappeared from the co-cultured population after 2 months of weekly passaging. Co-incubation of mESC with rat ECs without cell-to-cell contact did not result in the conversion of mESC into ECs. After co-cultivation of adult mesenchymal stem cells from human endometrium (eMSC) with pre-hepatocyte-like cells of human hepatocarcinoma Huh7 the resulting co-culture expressed mature liver markers (oval cell antigen and cytokeratin 7), none of which were expressed by any of co-cultivated cultures, thus proving that even an immature (proliferating) pre-hepatocyte-like line can induce hepatic differentiation of stem cells. In conclusion, we have developed conditions where long-term co-proliferation of embryonic or adult SC with fully or partially differentiated cells results in stem cell progeny expressing markers of target tissue. In the case of endothelial differentiation, the template population quickly disappeared from the resulted culture and the pure endothelial population of stem cell progeny emerged. This approach demonstrates the expected fate of stem cells during various *in vivo* SC-therapies and also might be used as an effective *in vitro* differentiation method to develop the pure endothelium and, potentially, other tissue types of desirable genetic background.

## Introduction

Cell therapies actively incorporate into modern medicine, not only for treating rare diseases but, in perspective, for wide-spread diseases such as diabetes mellitus (Memon and Abdelalim, 2020) and for aging treatment (Kovina et al., 2019), considering discoveries of new and productive stem cells (SC) sources: induciblepluripotent SC (iPSC), endometrial mesenchimal SC (eMSC) (Kovina et al., 2018). The key issue of successful cell therapy is the effective differentiation of stem cells into target cell type. Co-culture is one of the known differentiation strategies of modern science, in line with the addition of bioactive substances like growth factors, hormones etc. (Lagarkova et al., 2008; Kane et al., 2010; Kaupisch et al., 2012; Orlova et al., 2014), embryonic body manipulations (Li et al., 2008; Bahrami et al., 2012; Adams et al., 2013), incorporation of exogenous DNA, encoding induction/selection factors (Wang et al., 2007; Lindgren et al., 2015). The last approaches are expensive or raise some concerns about the safety of the clinical application (Kohn et al., 2003; Zaiss and Muruve, 2005; Incani et al., 2007) or result in heterogeneous cell population. For example, ECs (endothelial cells), derived from ESCs, contains 30–50% of non-ECs (Körbling et al., 2002; Park et al., 2009; Ikuno et al., 2017).

Co-culture as a differentiation approach imitates the differentiation of SC during the transplantation of bone marrow or solid organs. Though some authors do not believe in true differentiation of homed SC, but gender-mismatched donor/recipient patients reveal significant X/Y-chimerism in heart, lungs, liver, kidney, etc. (Theise et al., 2000; Körbling et al., 2002; Herzog et al., 2003; Kleeberger et al., 2003; Thiele et al., 2004; Minami et al., 2005). Even if *in vivo* chimerizm might be sometimes explained without differentiation, but at least *in vitro* the direct cell-to-cell contact during SC co-cultivation with differentiated cells promotes partial differentiation into myocytes, endotheliocytes, and hepatocytes with little or no fusion (Thiele et al., 2004; Wurmser et al., 2004; Xu et al., 2004; Lange et al., 2005), though no full conversion was yet shown. The cell-contact might work in combination with the secreted factors and growth factors from the medium (Bigdeli et al., 2009; Banerjee et al., 2011; Domev et al., 2012).

Earlier we have shown the adipose differentiation of mESC under co-cultivation with proliferating preadipocytes (Kovina et al., 2013). The data on the influence of co-culture on ES cell differentiation into endothelial lineage is largely lacking. EC is an essential cell type for tissue engineering, play a central role in vasculogenesis, angiogenesis and vascular pathogenesis (Hirashima et al., 1999; Poole et al., 2001) and is a promising therapeutic agent for the treatment of cardiovascular diseases such as myocardial infarction, peripheral vascular disease, and stroke (Sen et al., 2011; Shi et al., 2013).

The extremely low number of endothelial progeny of SC in the resulted co-culture (sometimes less than 1% of total cell number) is a common pattern for currently widely practiced co-culturing technique: short (4–20 days) one-round (with no passaging) co-incubation of stem cells with differentiated (feeder) cells sometimes treated with artificial proliferative blockers (Wurmser et al., 2004; Niwa et al., 2009).

Co-cultivation of ECs with SCs was also explored in 3D conditions, both in artificial scaffolds (Liu et al., 2019) and in cellular spheroids (Saleh et al., 2011). In both works endothelial cells presented the minority of the resulted heterogenous cell mass, possibly due to additional stimuli from the 3D matrix or organ-like asymmetrical stimuli in cellular spheroids.

Present work aimed to develop the effective co-cultivation method of inductive differentiation suitable for clinical applications, mimicking *in vitro* the symmetrical environment of proliferating SC, homed inside of homogenous tissue *in vivo*. We suppose that the continuous proliferation of diluted in homogenous tissue SCs increases the duration, symmetry and efficiency of the cell-to-cell contact, since the maximal acceptance to environmental stimuli might depend on cell cycle stage and number of cycles. We also tested, whether same approach can be suitable for different stem cell types, i.e., embryonic and adult stem cells, and for other types of differentiation (hepatic).

## Materials and Methods

### Experimental Procedure of Co-cultivation

Primary rat aortic ECs (passage 4) were kindly donated by Dr. Emil Martin (the Brown Foundation Institute of Molecular Medicine for the prevention of Human Diseases, University of Texas Health Science Center at Houston, Houston TX, United States). The cell suspension, frozen in serum-free medium with 10% (v/v) dimethyl sulfoxide, was thawed in 5-fold volume of complete endothelial medium composed of DMEM (Corning, Cat #10-013-CV) diluted 1:1 with F12K nutrient mixture Kaighn’s Modification (Gibco, Cat# 21127022) and supplemented with 10 mM HEPES buffer, 1% (v/v) of non-essential amino acids mixture (Gibco, Cat#11140050), 1% (v/v) of penicillin/streptomycin mixture (Corning, Cat#30002CI), 1 mM sodium pyruvate (Gibco, cat#11360070), 10% fetal bovine serum (Sigma, Cat#F2442), and 50 μg/ml Endothelial Cell Growth Supplement (BD Biosciences, Cat# 354006). Thawed cells were centrifuged for 1 min at 1,000 rpm, resuspended in the same medium, counted using Trypan blue staining, and plated into 6-well plates (Falcon) at concentration about 1 × 10^5^ cells/well. The medium was changed every third day. Cells were continuously passaged every 4–5 days at 80% confluency. Cells were trypsinized with 0.25% (m/v) trypsin solution (Gibco, Cat#25200056), and trypsinolysis was arrested by addition of 5-fold excess of the medium. The cell suspension was diluted 2–3-fold during each passage. The 6th and 7th passages were used for coculturing experiments.

Mouse embryonic stem cells (EZ-1), mESC, used in this study (derived from 129S1/SvImJ strain) were kindly provided by Dr. Eva Zsigmond (Institute of Molecular Medicine, University of Texas Health Science Center at Houston). The cells were initially grown on mitotically inactivated mouse embryonic fibroblasts (feeder layers), passaged two or three times, and subsequently transferred onto gelatin-coated plates to obtain feeder-free culture. The mESC were maintained in mESC medium composed from DMEM, 1% (v/v) non-essential amino acids mixture (Gibco, Cat#11140050), 10 mM HEPES buffer, 1 mM sodium pyruvate (Gibco, cat#11360070), 2 mM L-glutamine, 15% defined fetal bovine serum (HyClone, cat# SH30070.02), 1/75,000 dilution monothioglycerol (Sigma, Cat#M6145), and 1,000 U/ml leukemia inhibitory factor (MilliporeSigma, Cat#ESG1106). The whole procedure of mESC preparation takes 2–3 days, after which they are immediately used for co-culturing experiments.

#### Co-culture of mESC With ECs

For differentiation experiments, 10–1,000 μl of cell suspension at concentration about 100,000 mESC/ml were added to duplicate wells (6-well plates) with about 1 × 10^5^ recipient (template) ECs (at 1:100, 1:10, and 1:1 mESC:EC ratio) in EC medium or to the cell-free gelatin-coated wells used as mESC control in LIF-containing mESC medium (since in the EC medium mESC did not attach to the gelatin-coated plate). Inside of each experiment we used identical suspension of mESC, but different volumes: 10 μl for 1:100 ratio, 100 μl for 1:10 ratio and 1,000 μl for 1:1 ratio. It corresponds to 1,000 mESCs for 1:100 mESC:EC ratio, 10,000 mESCs for 1:100 ratio and 100,000 mESCs for 1:1 ratio. The amount of ECs in wells was constant and equals to 100,000 cells. For clarity, we have made changes in the section “Materials and Methods.”

Two to four wells were left without mESCs (endothelial control). The medium was changed every third day. Cells were continuously passaged every 5–7 days at 90–100% confluency until stable culture phenotype (usually 7–9 weeks). Cell suspension was diluted 4–6-fold during each passage. The whole co-culture procedure was repeated twice. Samples for immunocytochemical analysis were obtained by parallel passaging of cell aliquots into triplicate wells of 24-well plates with circular gelatin-coated glass coverslips on the bottom. One of triplicates was usually used for blind staining (no primary Ab control) to confirm the absence of non-specific secondary Ab binding. We skipped the photos of these negative controls, since we never got any positive signal in these settings.

Human endometrium mesenchimal SC, eMSC, were isolated from human menstrual blood according to the newly developed protocol (Kovina et al., 2018) and cultivated 2–3 passages before use in the MSC medium composed from 1õ1 alpha-MEM + Glutamax (Gibco, Cat#35571-028), 100 ME\ml Humulin (Eli Lilly), 1.6 nM Prednisolone Na phosphate (Biopharm Pvt Ltd., Cat#14592/02), 20 mM HEPES (Paneco, cat#Φ134), 20 ng/ml, fibroblast growth factor (Sigma, Cat#F0291), 10% fetal bovine serum (PAALaboratories, Cat#A15-101), 1% (v/v) Antibiotic-Antimycotic (Thermo Fisher Cat#15240062).

Hepatocellular human carcinoma cell line Huh7 was cultivated before use in the medium composed from DMEM/F12 + Glutamax (Gibco, Cat#35050061), 10% fetal bovine serum (PAA Laboratories, Cat#A15-101), 1% (v/v) Antibiotic-Antimycotic (Thermo Fisher Cat#15240062).

#### Co-culture of eMSC With Huh7

For differentiation experiments, 5–7 thousand cells of each culture, eMSC and Huh7 (25–50 μl of cell suspension at concentration 100,000–150,000 cells/ml) were added to duplicate wells of 96 well plate at 1:1 ratio or individually as pure eMSC\Huh7 control in DMEM\F12\FBS medium. This high ratio is necessary to use when template culture is immortal and has high proliferating rate during the whole differentiation experiment. The medium was changed every third day. After 2 weeks of cultivation the confluency was reached and we terminated the co-cultivation. The medium was removed and, 100 μl of cold methanol was added to each well and plates were frozen till immunocytochemical analysis. The whole co-culture procedure was repeated twice with the same results. A few wells of the plate were used for blind staining (no primary Ab control) to confirm the absence of non-specific secondary Ab binding. We skipped the photos of these negative controls, since we never got any positive signal in these settings.

### Immunocytochemistry

#### Primary Antibodies

We have used:

mouse anti-rat endothelial cell antigen 1 antibody (anti-RECA-1 Ab) produced by GeneTex (Monoclonal, Clone Name RECA-1, Reactivity: Rat, Cat#GTX54491),
rat anti-mouse endothelial CD102 antibody (Monoclonal, Clone 3C4, BD Biosciences, reactivity: mouse, Cat#550544),
rat anti-mouse endothelial CD29 antibody (Monoclonal, clone KMI6, BD Biosciences, reactivity: mouse, Cat#55874),
rat anti-mouse endothelial CD31 antibody (Monoclonal, clone MEC13.3, BD Biosciences, reactivity: mouse, Cat#553370),
mouse anti-human monoclonal anti-cytokeratin 7 antibody (Monoclonal, clone OV-TL12/30, ThermoFisher, reactivity: mouse, human, Cat#CN180234), (Maehara et al., 1996),
rat anti mouse\human anti-A6 antibodies (Monoclonal, reactivity: mouse, human). Were produced and kindly donated by Prof. N. V. Engelgard (PMID: 2095484).

#### Secondary Antibodies

We have used:

Alexa Fluor 488 goat anti-mouse IgG (Polyclonal, Molecular Probes, Cat#A11001),
Alexa Fluor 594 goat anti-rat IgG (Polyclonal, Molecular Probes, Cat#A11007),
Alexa Fluor 488 goat anti-rat IgG (Polyclonal, Abcam, Cat#ab150157),
DAPI (4′,6-diamidino-2-phenylindole) was purchased from Sigma (Cat# D9542).

#### Immunofluorescence

##### Staining of Surface CD-Antigens

Conventional protocols for cell fixation with paraformaldehyde or other denaturing agents (methanol, acetone) prior to antibody staining inhibited the binding of the antibodies to the cell surface. Therefore, for staining of CD-antigens the conventional protocol (Sen et al., 2011) for a half-native staining was modified as follows. Gelatin-coated glass slips (∼100 mm^2^) with grown cell monolayer were inverted onto 10 μl drops of primary Ab solution, diluted 2–5-fold by 1% (m/v) BSA (50–100 μg/ml final Ab concentration) in PBS (pH 7.4; Gibco, cat# 10010023), and placed in a humid box at 37°C for 30–60 min. Each coverslip was then washed gently in the staining rack cassette filled with 1% (m/v) BSA in PBS (∼100 ml) for 5–15 min. The whole cassette was very gently transferred into fresh PBS (100 ml) for 5 min, then into 4% (m/v) paraformaldehyde in PBS for 10 min, then into fresh PBS for 5–10 min, and then into 1% (m/v) BSA in PBS for 2 min. The coverslips were then inverted onto 10 μl drops of secondary Ab solution diluted with 1% (m/v) BSA in PBS 100–300-fold (7–20 μg/ml final Ab concentration). Secondary Ab solution contained 4 μM DAPI (2 μg/ml) where indicated. Slides with secondary Ab were placed in a humid box at 37°C for 30–60 min. Then the coverslips were put into a washing rack cassette with PBS for 1 min, and then the whole cassette was transferred into 1% (m/v) BSA in PBS for 15 min. The coverslips were then inverted onto 5 μl drops of mounting medium (MilliporeSigma, cat#34578920ML), and observed using an Optiphot microscope (Nikon, Inc., Melville, NY) with air objectives (magnification is indicated on the figures), using default gain settings. Images were recorded using a MagnaFire color digital camera (Optronics, Goleta, CA), exposition time is indicated on the figures. The strategy to select the optimal exposition time is illustrated by Supplementary Figure 1.

##### Staining of Intracellular and Intranuclear Antigens Cytokeratin 7 and Oval Cell Antigen A6

The cells were treated with cold-frozen methanol (−20°Ñ) and left at below −5°Ñ for 30 min under parafilm, washed with PBS + 0.1% (v/v) tween-20 for 10 min, washed with 10% FBS + PBS + 0.1% (v/v) tween-20 for another 10 min, and stained overnight with added primary antibodies (anti-Cytokeratin 7 and antiA6) in final concentration 1:200 (diluted with PBS + 0.1% (v/v) tween-20). The next day the cells were washed twice with 10% FBS + PBS + 0.1% (v/v) tween-20 for 10 min, the washed with 2% (m/v) BSA + PBS + 0.1% (v/v) tween-20 for 10 min, and stained overnight with added secondary antibodies in final concentration 1:500 in 10% FBS + PBS + 0.1% (v/v) tween-20. The third day the cells were washed twice with 10% FBS + PBS + 0.1% (v/v) tween-20 for 10 min, then washed with 2% (m/v) BSA + PBS + 0.1% (v/v) tween-20 for 10 min. The images were taken with fluorescent microscope NIKON Eclipse TE2000-U with air objectives, using default gain settings, while exposition time and magnification are indicated on the figures.

#### The Digital Processing of Fluorescent Photographs

For quantitative estimate of the difference in fluorescence between pure cultures and resulting co-culture, their images were processed with the program ImageJ as follows. Initial fluorescent photographs were split to color channels, the green 488nm or red 594nm channel were taken for further processing as indicated. The optimal value of signal threshold was selected for the green-channel triplet as it is described in Supplementary Figure 2A. The summary surface area of open spots was calculated for each frame within the duplet (Supplementary Figure 2D) or triplet (Supplementary Figure 2C). The mean values and standard deviations of spot areas were calculated after processing of a few typical photograph triplets of eMSC:co-culture:Huh7 as it is shown by Supplementary Figures 2B,C.

## Results

### Endothelial Differentiation

**FIGURE 1:**
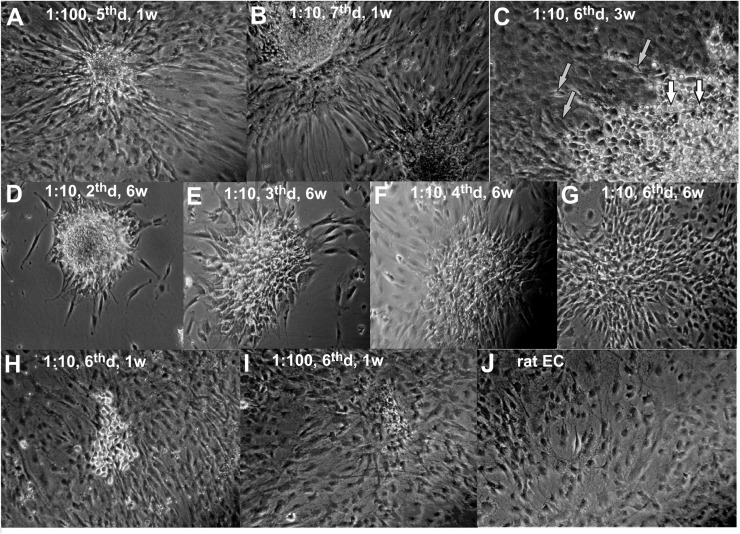
The mESC + rEC co-culture at different stages **(A–I)** and rEC monoculture **(J)**. The mESC:rEC ratio, the day after the last passage (d) and the age of the co-culture in weeks (w) are given on the photos. **(A,B)** Typical star like colonies of mESCs formed to the 5th–7th day after passaging with rEC. **(C)** Sector of typical mESC colony at higher magnification. **(D–F)** Typical development of mESC colony after passaging with rECs. **(G–I)** Terminal stages of mESC colony development when it disappears (usually to the 6th day after last passaging). **(J)** Original primary rat endothelial culture. **(A,B,D–G)** Magnification 100x. **(C,H–J)** Magnification 200x.

In order to investigate the fate of mESC in long-term co-culture with EC we performed co-culture experiments with different initial mESC:EC ratios. Two types of mESC development were observed: colony-forming symmetrical proliferation (self-renewal or central 3D-growth) and peripheral “beam-growth,” where cell beams intrude into surrounding EC monolayer (Figures 1A,B). It is seen, that SC colonies crosstalk with endothelium. The non-random orientation of ECs toward the SC colonies confirms active cell communication. The details of the “beam-growth” at higher magnification are shown in Figure 1C. Small round central cells (Figure 1C, lower right corner, white arrows) representing the typical morphology of undifferentiated mESC, shown also in Figures 1A,B, convert into spindle-shaped cells (gray arrows), morphologically identical to ECs, through intermediate stages of radial growth. Squeezing into the surrounding monolayer in a beam-like manner they finally become indistinguishable from the surrounding cells by morphology but keep the radial orientation. These two types of growth cause the special star-like shape of mESC colonies within endothelial culture (Figures 1A,B,F). The central growth prevails when the cell density is low (Figure 1D). Peripheral “beam” growth occurs when proliferating ECs approach and surround mESC colonies (Figures 1E,F). If a mESC colony is not too large at this moment, it disappears before the next passage, “dissolving” into the endothelial monolayer (Figure 1G). Colonies that did not “dissolve” start the “stars” of the next generation after trypsinolysis and passaging, after which the contact surface between lineages increases, stimulating further differentiation (beam growth) and inhibiting the central colony growth. Therefore, the increase or decrease in the size and number of mESC colonies during the course of cultivation depends on the initial mESC:EC ratio and the passaging frequency. For practical use, to produce the maximal amount of pure population of differentiated SC progeny, one should find the optimal cell ratio and then regulate the colony growth by the passaging frequency, thus approaching conditions under which the number and the size of mESC colonies whether slowly decreases (to produce the homogenous differentiated culture) or remains constant (to maintain the mixed permanent culture, which can be used for permanent production of unlimited amount of differentiated cells).

We found that the optimal mESC:EC ratio is 1:10 for the passaging frequency of once every 5–7 days, and under these conditions the number of mESC colonies gradually decreases. Seeding mESC to ECs at 1:10 results in the formation of star-like mESC colonies on the day of confluent monolayer formation (4–6th day after each passaging, Figures 1A,B,F). The continuous passaging of 1:10 co-culture during 1.5–2 months results in a gradual decrease of number and size of mESC colonies and eventually to their total disappearance (Figures 1D–G). The terminal development stages before the disappearance of mESC colonies are shown in Figures 1H,I. The derived cells (Figure 1G) are not visually distinguishable from the initial rat EC population (Figure 1J), though they may still keep the radial orientation.

**FIGURE 2:**
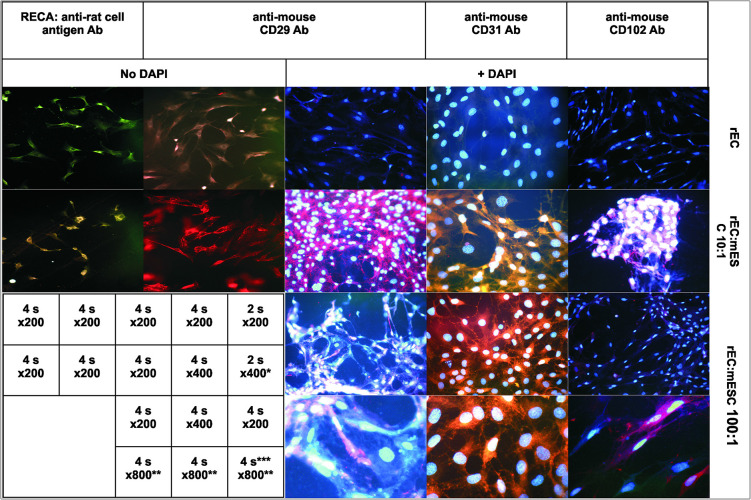
Fluorescent immunostaining of rat endothelial cells (rEC) alone (upper panels) or co-grown with mESC at initial ratio 10:1 (second row) or 100:1 (two lower rows). Primary Ab: mouse anti-rat-RECA-1 (left column); rat anti-mouse CD29 (2nd and 3rd column); rat anti-mouse CD31 (4th column); and rat anti-mouse CD102 (right column). Secondary Ab: goat anti-mouse Alexa Fluor 488 (left column); and goat anti-rat Alexa Fluor 594 (all other). Columns 3rd-5th: DAPI, 2 μg/ml. Magnification and exposition are indicated on the table insert. *The photo was obtained at magnifications x200 and enlarged digitally 2-fold. **Photos in the lowest row were obtained at magnifications x200, x400, x400, respectively, and enlarged digitally 4 or 2-fold to obtain x800 final magnification. ***Brightness\contrast settings were adjusted to underline the difference between colored and uncolored cells on the same picture.

To determine whether the resulting cell population consists of the mouse or rat progeny cells and whether it expresses the endothelial markers, we stained the cells with antibodies against the specie-specific endothelial markers: rat RECA-1, mouse CD29, mouse CD31 and mouse CD102 (Figure 2).

Rat ECs displayed good signals (green fluorescence) with anti-RECA-1 antibodies (the upper photo of the first column, Figure 2) while giving no specific response to anti-mouse CD29, CD31, and CD102 antibodies (the upper row of 2nd–5th columns, Figure 2). The difference in signal intensity between monoculture and co-culture is 5–7 folds as it is shown in Supplementary Material via the spot-reading program ImageJ (Supplementary Figure 2D).

The final co-culture populations showed no response to anti-RECA-1 antibodies (the lower photo of the first column, Figure 2) while exhibiting strong red fluorescence with all anti-mouse antibodies (the second row of 2nd–5th columns, Figure 2). Therefore, 2 months long exponential co-proliferation of mESC mixed in initial ratio 1:10 with rat ECs results in the cell population expressing only mouse endothelial surface markers (CD29, 31,102), whereas rat endothelial cell antigen disappeared from the co-cultured population.

At higher seeding ratios (rarely for 1:10 seeding and always for 1:1 seeding) some colonies exhibited uncontrolled central 3D-growth that was not stopped by confluence or passaging. Some of these 3D-colonies eventually formed beating bodies unless replated. After passaging, these enormous colonies lose the potential to differentiate into homomorphic endothelial culture since the number of cells within colonies overwhelms the total number of template ECs, and the passaging of such overgrown co-culture leads to a heterogeneous cell population which loses endothelial morphology (not shown). This is easily prevented by maintaining a low ratio of mESC to ECs, i.e., by using the low initial ratio of SC to template culture (1:10 or lower) and by frequent passaging (at least once each 5–7 days).

At lower ratio of mESC to EC (1:100 and lower) mESC differentiated too rapidly without sufficient proliferation, and the resulting culture contained up to 10% rat cells not stained by anti-mouse antibodies (two bottom rows of 3rd–5th columns, Figure 2, unstained cells are seen). In the co-culture resulting from 1:1,000 seeding we did not find any well stained mouse cells. At this high dilution, SCs apparently differentiate into ECs immediately without much proliferation and therefore lose their progenitor’s potential to outnumber surrounding cells. This conclusion is supported by the fact that we did not observe any “stars” in 1:1,000 seeded co-cultures even for initial passages, and by the fact that these co-cultures became extinct after 7–8 passages in co-culture, which is about the same time as pure endothelial cultures reach the Hayflick limit (13–15 passages total). Therefore, the success of effective differentiation with no template cells in resulting culture strongly depends on the initial ratio of seeded SC to allo-(xeno-)geneic template cells.

**FIGURE 3:**
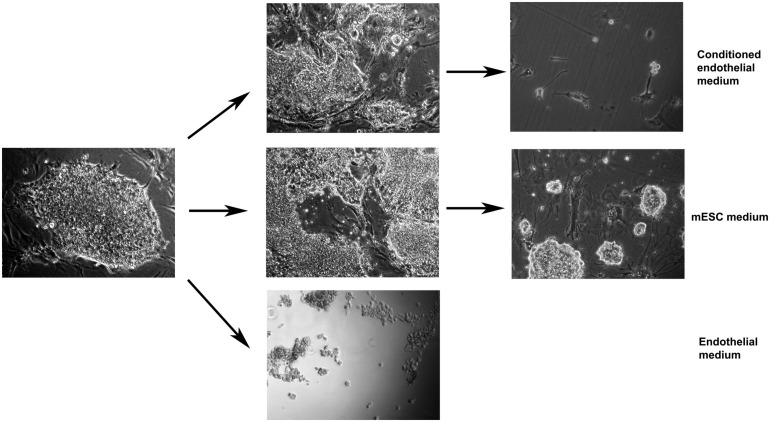
Mouse embryonic stem cells alone grow well in the mESC medium (middle row), do not attach/survive in fresh endothelial medium (lowest photo), attach but do not proliferate in conditioned endothelial medium (upper row). Arrows designate single passaging events (dilution of 5–7x). Magnification: 40x (lowest photo), 100x (other photos).

To address the question of whether the cell-to-cell contact is necessary for endothelial differentiation or the co-cultured ECs produce some soluble components which induce the endothelial differentiation, we separated co-cultured cells either using membranes (FALCON, 0.4 μm pore size) or used the preconditioned endothelial medium (24 h preincubated with ECs only) for further mESC culturing. Both methods gave similar results. A healthy monolayer of mESCs on the gelatin coated plate was established before EC conditioned media was applied. mESC survived in the conditioned EC medium after replating and attached again (Figure 3 upper left), however, they did not grow, and, therefore, disappeared after two consecutive diluting passages (Figure 3 upper right),. In the fresh unconditioned EC medium mESC did not survive replating since did not attach to the gelatin-coated plate at all (Figure 3, the lowest photo). Therefore, only soluble factors of the medium, including unstable factors, are not sufficient for successful proliferation-dependent differentiation.

**FIGURE 4:**
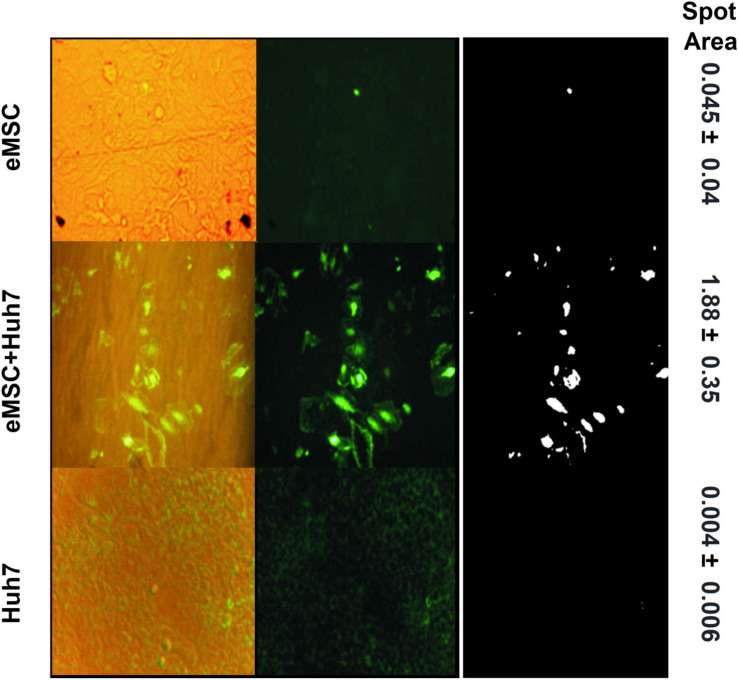
Double staining of eMSC + Huh7 co-culture and each monoculture for periductal liver antigens associated with pre-hepatocytes: oval cell antigen A6 (nuclear localization) and cytokeratin 7 (cytoplasmic localization). Upper row: eMSC (4th passage), middle row: eMSC after 2-week coculturing with Huh7; lowest row: human hepatocellular carcinoma cell line Huh7 alone. Left column: overlap of phase contrast photographs and fluorescent photographs; middle column: fluorescent photographs; right column: the ImageJ-derivative of the middle column. The details of quantitative analysis of fluorescent areas are given in the section “Materials and Methods” and in Supplementary Figure 2. Magnification: 200x, exposition\brightness settings are the same in each vertical triplet (column).

We also applied the co-cultivation method for adult stem cells from human endometrium (eMSC), differentiating them toward hepatic lineage via co-cultivation with hepatocarcinoma Huh7 (Figure 4). Individual cultures of eMSC (upper row of Figure 4) and Huh7 (lowest row of Figure 4) give very weak or no signal of cytokeratin-7 and A6 (oval cell antigen). After co-cultivation, the very strong signal of both antigens, CK7 (cytoplasmic localization) and A6 (nuclear localization) emerges in the resulting co-culture (middle row of Figure 4). Fluorescent signal from co-culture, calculated as fluorescent spot area (1.88 **±** 0.35, see the right column of Figure 4), was at least 40 times stronger, than from eMSC (0.045 **±** 0.04, compare the middle and the upper rows of Figure 4); and ∼400 times stronger, than from Huh7 (0.004 **±** 0.006, compare the middle and the lowest rows of Figure 4). The details of quantitative analysis of fluorescent areas are given in the section “Materials and Methods” and in Supplementary Figure 2.

Unlike the final culture resulting from the first co-cultivation set (mESC + EC), the final culture of the second set (eMSC + Huh7) still contains template Huh7 cells, since they are immortal.

## Discussion

We have found that serial passaging of co-cultured mESC and rat EC at initial ratio 1:10 results in homogenous culture expressing mouse endothelial markers and no rat endothelial markers. This means that:

First, under these conditions all mESC converted into ECs, since all visualized cells expressed the mouse specific endothelial antigens CD102, CD31, and CD29, while rat endothelial marker RECA fully disappeared. While rat markers were reported to be highly specific for rat endothelial tissue (Niwa et al., 2009), the tissue specificity of mouse markers is not absolutely strict. However, their weak non-specific expression was shown for tissues morphologically distinct from ECs, that is, leukocytes and smooth muscle cells (Saleh et al., 2011). Cells of this morphology, or of mESC morphology, were not observed in our co-cultures. Thus, the strong signal of several endothelial markers and morphological homogeneity of the resulting co-culture confirms its endothelial nature.

*Second*, the cells in the resulting population are of mouse origin. The progeny of seeded mESC overgrew rat ECs until the apparent disappearance of the latter. Antibodies we used were specie-specific without cross reactivity toward rat cells (compare Figure 2 upper row with the second row), in full accordance with published data (Niwa et al., 2009; Kovina et al., 2018). This confirms effective conversion of the initial rat endothelial culture into mouse ESC-derived progeny via continuous co-growth. The overgrowth of rat cells by mouse cells with practically full replacement of initially prevalent template cells by mESC progeny may result from the higher proliferation rate of mESC and their progeny and\or from eventual replicative senescence of primary rat endotheliocytes once they reached the Hayflick limit. Indeed, at the end of an experiment (about 13-15 passages total) the pure rat endothelial culture looked unhealthy in most of the control plates (not shown) and ceased to expand.

Unlike the first co-cultivation set mESC + EC, the second set of co-cultivated cultures (eMSC + Huh7) does not result in the homogenous culture, since template culture Huh7 is rapidly proliferating and it is immortal, therefore it cannot be exhausted. To get the homogenous culture of prehepatocytes one needs to use normal hepatic tissue as a template, though at present it is difficult to maintain mature hepatocytes *in vitro* for a prolonged period of time.

The mechanism of differentiation via co-cultivation is unlikely to be fusion. Since: (i) the nuclei of the resulting and initial cells look quite similar (Figure 2), independently of the presence or the absence of mouse or rat cell surface antigens, while nuclei of fused cells were shown to be much larger than nuclei of non-fused cells (Willenbring et al., 2004); (ii) the resulting cells do not express the surface antigen of the initial culture RECA-1; (iii) fusion was carefully excluded in earlier studies for similar co-cultivation sets by single-cell PCR (Kögler et al., 2004) or by chromosome imaging (Wurmser et al., 2004).

What are the major signals for template-directed differentiation during cell co-proliferation? In our work we have shown that cell-to-cell contact is essential for it, since we did not observe any differentiation in the control experiments with conditioned medium or with the cells separated by membrane, which confirms the conclusion of other authors (Li et al., 2004; Shen et al., 2004; Wurmser et al., 2004) that both soluble factors and cell-contact signals are necessary for directed differentiation. These results agree with previous reports that soluble factors of the preconditioned medium cannot promote the endothelial differentiation (Xu et al., 2004; Shi et al., 2013; Ikuno et al., 2017), and direct cell-to-cell contact is necessary. In addition, we have shown that the effective differentiation needs significant time over repeated passaging cycles. Repeated passaging stops central 3D-growth of a SC colony, segregating it during each trypsinolysis onto single SC or small SC aggregates. This reopens SC surfaces to inductive signals from ECs, promoting the integration of SC progeny into template culture via peripheral growth and differentiation. AT this stage of our research we cannot distinguish between possible types of contact inductors. IT could be junctions or matrix or nanotubes with microRNA or even directed mechanical (lateral) pressure, stimulating conversion of round stem cell into elongated endothelial cell, as can be concluded from the perfect ordering of radially oriented newly differentiated EC (Figures 1A,B). Mechanical pressure (rhythmic stretch) was shown to be sufficient for cardiomyogenic differentiation of SC (Huang et al., 2012).

The condition of continuous co-proliferation enabled by repeated passaging was absent in earlier studies: to avoid 3D-growth of SC, analogous to teratoma formation, a frequent complication of embryonic SC therapy, investigators usually co-incubate cells during only a single passaging cycle at low ratio of SC to template cells. Passaging the co-culture each time when SC colonies start central growth gives a new differentiation chance to the SC in the middle of the SC colonies. This is also the difference of our work from 3D technologies, which do not give monocultures, but rather organ-like structures (Incani et al., 2007; Wang et al., 2007). Adjusting the initial ratio and the frequency of passaging, we regulate the number and size of star-like SC colonies in the continuous co-culture, not allowing them to disappear too quickly (before all the template cells would be replaced by SC progeny) or to overgrow.

Discussing the implementation of the suggested here technique into current industry standards, we can make simple calculations: during 7–9 weeks of co-cultivation (about 10 passages) at the passaging diluting of 4 to 6 fold the whole procedure can give up to ∼5^10^ = ∼10^7^ fold multiplication of the initial cell number (let say 10^5^ cells), resulting in 10^12^ cells, which is comparable to the amount of the cells in human body. Current protocols of human EC therapy requires from 1.5 × 10^6^ (Numa et al., 2020) to 2 × 10^9^ cells (calculation based on human/mouse weight ratio) (Poulos et al., 2015). Since it could be difficult to produce such amount of cells in standard laboratory 2D plates (2 × 10^9^ cells would occupy about 2 m^2^), the industrial 3D cell culture systems should be used, and appropriate bioprocess screening technologies applied (Abraham et al., 2018).

Besides 100% endothelial differentiation of mESCs without traces of xenogeneic template in the resulted co-culture, we obtained efficient pro-hepatic differentiation of adult endometrial MSCs on the immortal template cell line Huh7. eMSC are multipotent stem cells with broad spectrum of described differentiation potential: chondro-, adipo-, osteo, neurogenic etc. (Anisimov et al., 2013; Kovina et al., 2018). After co-cultivation eMSC started to express cytokeratin-7 and A6 (oval cell antigen), the markers of hepatic periductal area and hepatic stem cells (Maehara et al., 1996; Cabibi et al., 2003; Fougère-Deschatrette et al., 2006; Zhang et al., 2009; Matsukuma et al., 2012). Thus, we have shown that the contact differentiation phenomenon works also for pro-hepatic differentiation. However, for clinical use primary (untransformed) pre-hepatocytes must be used instead of Huh7. For example, xenogeneic pre-hepatocyte culture obtainable from neonatal animal liver or even from the liver, regenerating after partial hepatectomy, is similar to Huh7 in many aspects including ability to proliferate quickly and to express the same embryonic markers like AFP (Kawai et al., 2001; Gong et al., 2013) and Ki67 (Gerlach et al., 1997; Jabari et al., 2009). Therefore, such non-transformed proliferating pre-hepatocytes may serve as a safe self-disappearing xeno-template to produce the hepatocytes of the same genetic background as a patient, from his own MSC. There are still some ethical concerns about usage of animal tissues for clinics, however these concerns might disappear in the future after improved reliability of diagnostics methods for animal-derived pathogens.

Concluding, the successful template differentiation was confirmed on two different co-cultivation pairs, and, therefore, is expected for other cell pairs as well. This method, spread on wide variety of mortal template lineages (especially those of therapeutic significance: hepatocytes, cardiomyocytes, chondrocytes, beta-cells, etc.) is a promising approach in the future regenerative medicine because efficiency of differentiation via continuous co-cultivation reaches 100% as without cell-sorting techniques and concomitant losses as well without expensive growth factor cocktails. The need for template cells is not a shortage for human application, since the xenogeneic template might be used to generate fully syngeneic human transplant from human SC.

## Data Availability Statement

The original contributions presented in the study are included in the article/Supplementary Material, further inquiries can be directed to the corresponding author/s.

## Author Contributions

MVK contributed to conception and design, collection and assembly of data, and manuscript writing. TD contributed to data analysis and interpretation manuscript writing and administrative support. MEK contributed to consultations and technical supervision in the eMSC research. SY contributed to the statistical analysis of ImageJ-derivatives of initial fluorescent photos. YK contributed to data analysis and interpretation and manuscript writing. All authors gave final approval and agreed to be accountable for all aspects of the work.

## Conflict of Interest

The authors declare that the research was conducted in the absence of any commercial or financial relationships that could be construed as a potential conflict of interest.
