# Supplementary material for: Co-growth of Stem Cells With Target Tissue Culture as an Easy and Effective Method of Directed Differentiation
Source: Front Bioeng Biotechnol. 2021 Jun 16;9:591775. doi: 10.3389/fbioe.2021.591775 (PMC8242343; doi:10.3389/fbioe.2021.591775)

Supplemental material to the article:

Co-growth of stem cells with target tissue culture as an easy and effective method of directed differentiation.

Marina Valentinovna Kovina, Tatyana Gennadievna Dyuzheva, Mikhail Evgenievich Krasheninnikov, Sergey Alexandrovich Yakovenko, Yury Mikhailovich Khodarovich

Supplemental figure 1. Different exposition times and different magnifications, supplementing the Fig. 2 of the main manuscript. **A,** antiCD31 staining; **B**, antiCD29 staining; **C,** antiCD102 staining. Exposition time and magnification are indicated on the figures.

Supplemental figure 1A: antiCD31 staining, magnification x400

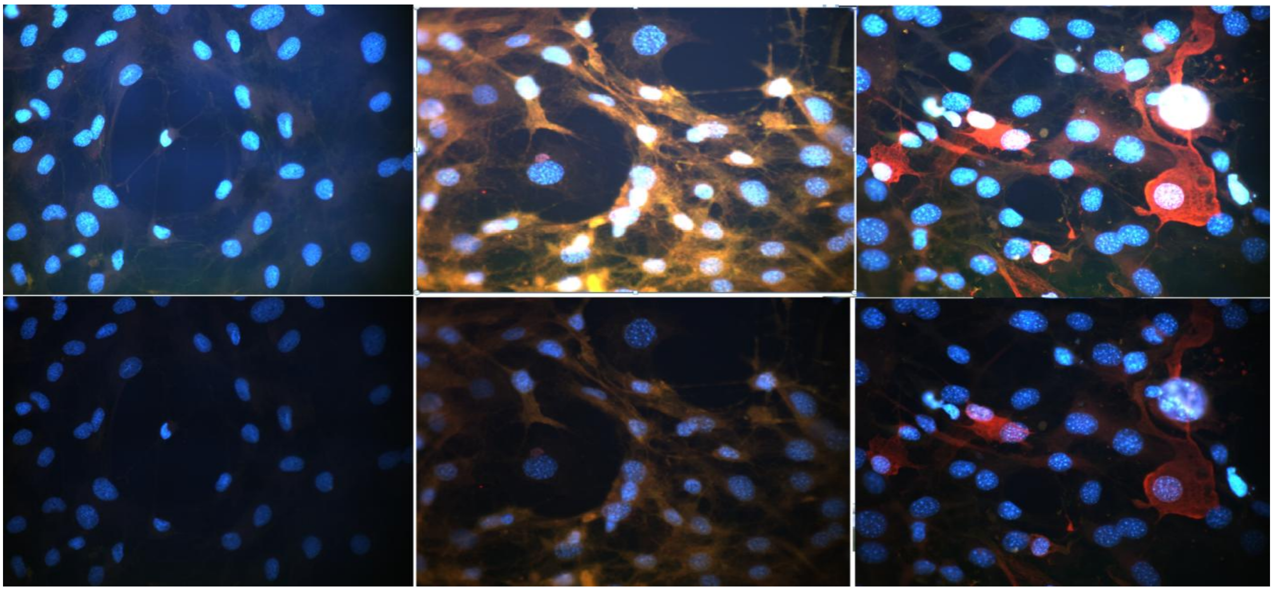

Supplemental figure 1B: antiCD29 staining, magnification x200

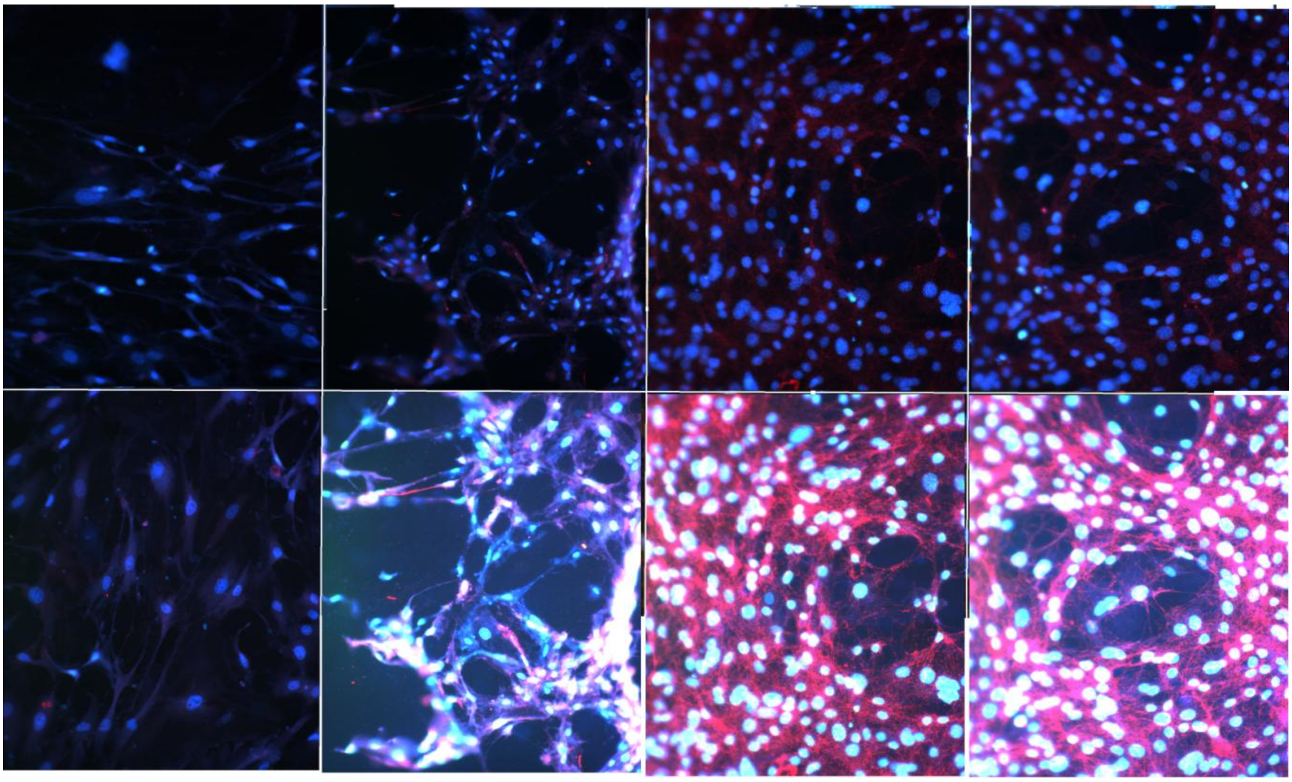

Supplemental figure 1C: antiCD102 staining, exposition time: magnification

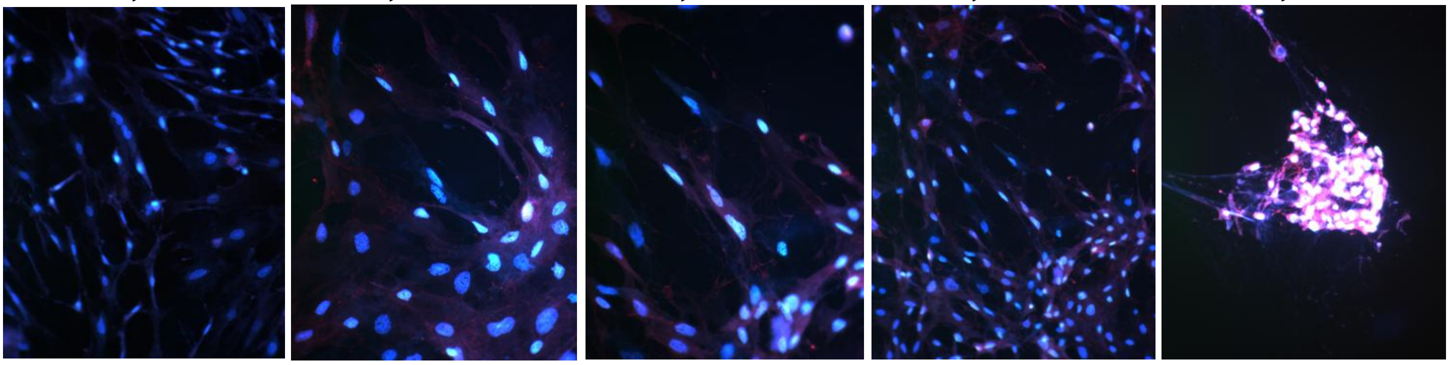

All photographs in the same rows were done in the same day with the same acquisition settings except the settings indicated on the label. Several pictures show heterogenous cell content, where some cells are non-colored, indicating no Ab binding (no primary, neither, therefore, secondary Ab), while other cells in the same photo are well stained.

For example, the same day at the same conditions as we tested CD31 expression on endothelial+mESC co-cultures, we obtained convincing heterogenous staining of simultaneously treated co-cultures mESC+adipocytes (last panels of supplemental figure 1A, SFig. 1A).

Another heterogenous staining, proving the absence of non-specific binding of secondary Ab, is seen on middle panels 100:1 of SFig. 1B and 1C, where a few non-colored cells are present on each 100:1 photograph. Non-colored cells work as the negative internal control for the absence of non-specific secondary Ab binding.

Beside internal control, the separate non-co-culture control (initial rat endothelial cells) represents the control for the absence of non-specific secondary Ab binding (all left panels of SFig. 1).

---

*Adipose differentiation via co-culture is described in Kovina M.V., Zilberman L., and Khodarovich Y. M.. (2013) «Differentiation of pluripotent stem cells during their cocultivation with adipocytes”. *Biomedicine of RAMS*, №4, 107-113

Supplementary Figure 2. Quantitative analysis of fluorescent areas of (eMSC:co-culture:Huh7) photographs, supplementing the Fig.4 of the main manuscript. **A,** The strategy to find the optimal signal threshold for digital processing of immunofluorescent staining results. Fluorescent spot areas are calculated with the program ImageJ and are given in the table above images for each (**eMSC:co-culture:Huh7)** triplet; **B,** Additional triplets of (eMSC:co-culture:Huh7) photographs, which were used for the calculation of mean values and standard deviations of fluorescent spot areas; **C,** The same as SFig.2B, but after processing at the optimal threshold and calculating spot areas, their mean values and standard deviations (the table above images).

Supplementary Figure 2A.

**Fluorescent spot areas**		
**0.014**	**0.053**	**0.878**
**0.945**	**2.584**	**6.503**
**0**	**0.003**	**9.646**

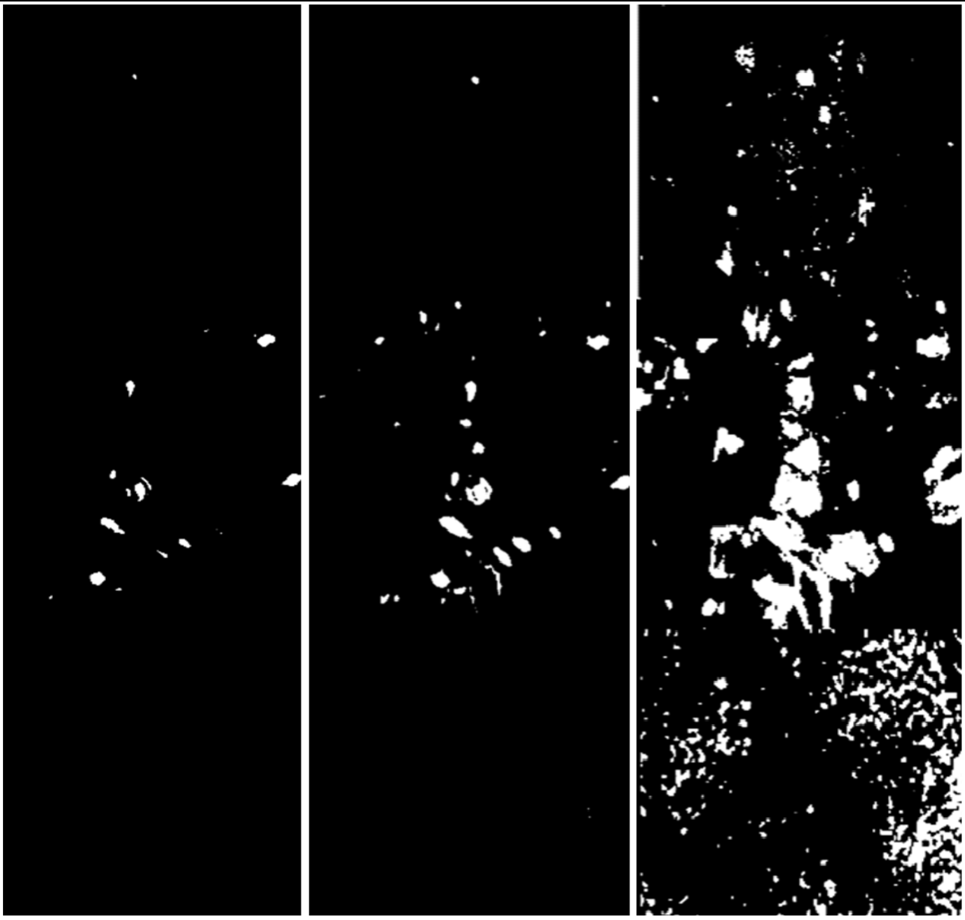

High threshold **Optimal threshold** **Low threshold**

At the threshold which is too high (left column) some of specifically stained cells disappear. At the threshold which is too low (right column) the unspecific signal is magnified unacceptably in control panels (monocultures). The middle column illustrates the optimal signal threshold, used for processing of the Fig.4B in the main manuscript.

Supplementary Figure 2B. Additional triplets of (eMSC:co-culture:Huh7) photographs, which were used for the calculation of mean values and standard deviations of fluorescent spot areas.

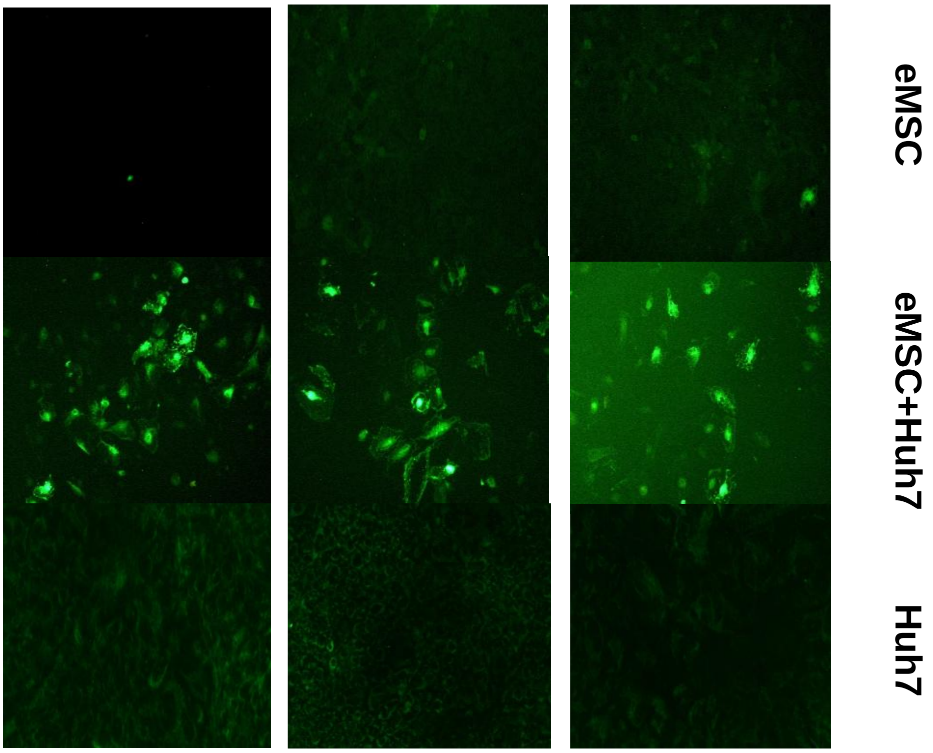

Supplementary Figure 2C. The same as SFig.2B, but after processing at the optimal threshold and calculating spot areas, their mean values and standard deviations (the table above images)

**Fluorescent spot areas**			**Mean area**	**Standard deviation**
**0.013**	**0.026**	**0.096**	**0.045**	**0.04**
**1.948**	**1.506**	**2.210**	**1.88**	**0.35**
**0.000081**	**0.000080**	**0.012**	**0.004**	**0.006**

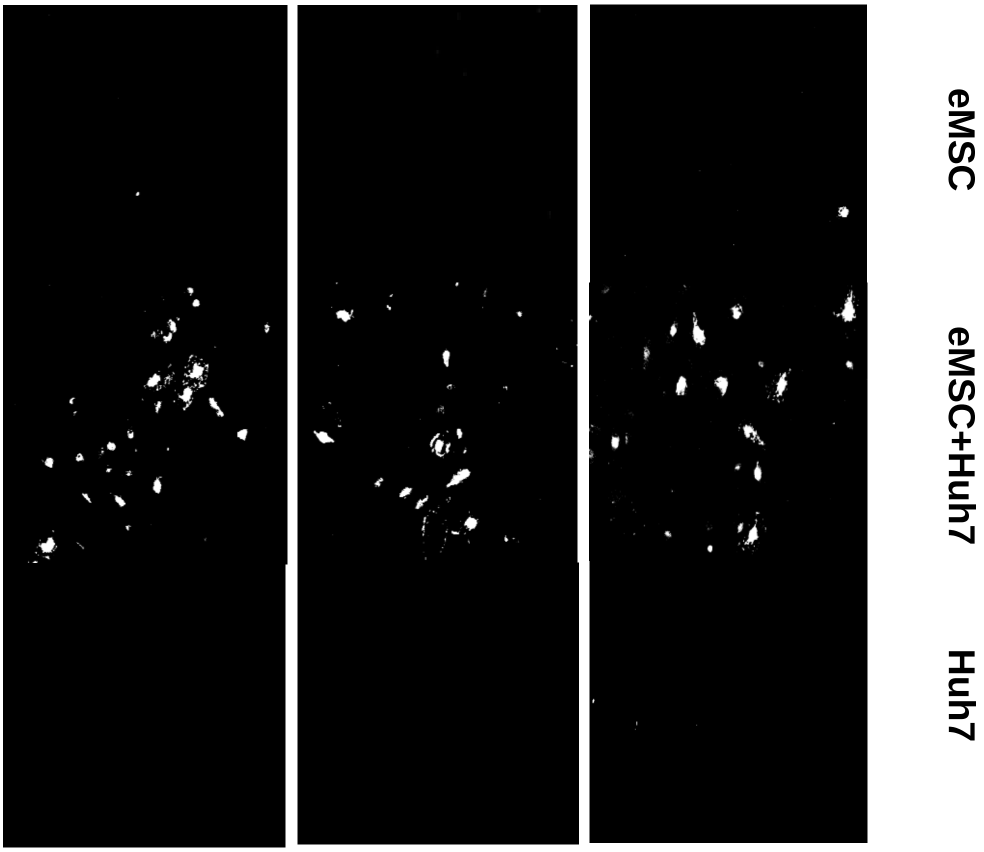

Supplementary Figure 2D. The same processing as above was done for two left columns of the figure 2 from the main text (here the bottom half of the figure). Fluorescent spots are selected in green channel on the left or in red channel on the right and are given as white on the upper half of the figure. Spot areas are calculated with the program ImageJ and are listed in the table. The difference between spot areas from co-culture and monoculture is 5 to 6 fold, which is significant.

**Fluorescent spot areas**	
**Green**	**Red**
**0.322**	**0.772**
**0.056**	**3.367**

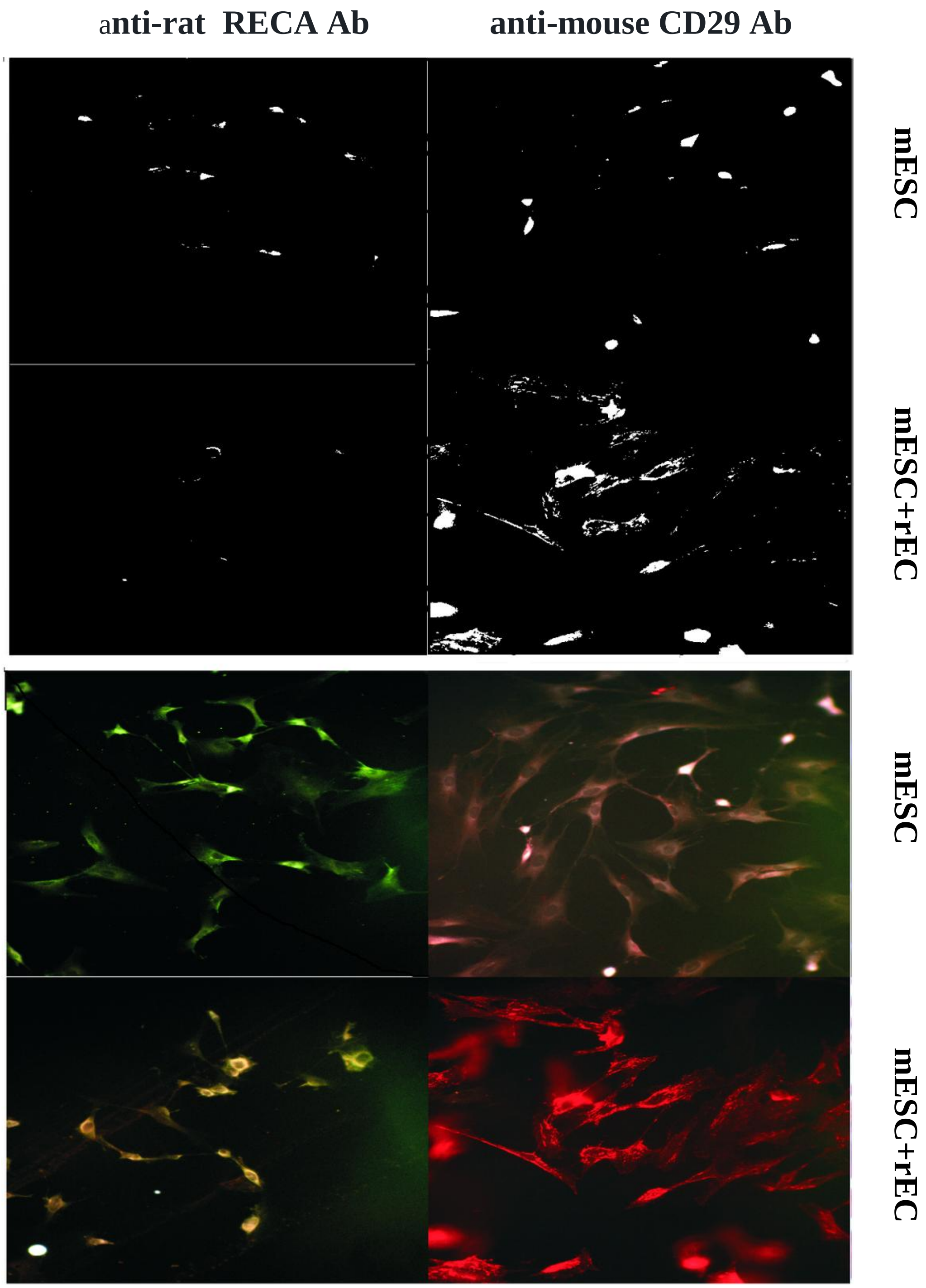

Supplement: Supplementary file 1 [file Data_Sheet_1.pdf]
